# A Composite Score for Predicting Vertical Transmission of Hepatitis C: A Multicenter Study

**DOI:** 10.3390/pathogens13010045

**Published:** 2024-01-03

**Authors:** Paul Wasuwanich, Joshua M. So, Brett Presnell, Wikrom Karnsakul, Robert S. Egerman, Tony S. Wen

**Affiliations:** 1 University of Florida College of Medicine, Gainesville, FL 32610, USA; p.wasuwanich@ufl.edu (P.W.); joshua.so@ufl.edu (J.M.S.); 2 Department of Statistics, University of Florida, Gainesville, FL 32611, USA; presnell@ufl.edu; 3 Division of Pediatric Gastroenterology, Hepatology, and Nutrition, Department of Pediatrics, Johns Hopkins University School of Medicine, Baltimore, MD 21205, USA; wkarnsa1@jhmi.edu; 4 Division of Maternal-Fetal Medicine, Department of Obstetrics & Gynecology, University of Florida College of Medicine, Gainesville, FL 32610, USA; egerrs@ufl.edu

**Keywords:** pregnancy, antiviral agents, public health, lymphocytes

## Abstract

Background: Prevention of the vertical transmission of the hepatitis C virus (HCV) presents an obstetric challenge. There are no approved antiviral medications for the treatment or prevention of HCV for pregnant patients. Objective: We aimed to create a composite score to accurately identify a population of pregnant patients with HCV who have high potential for vertical transmission. Study Design: In a retrospective, multicenter cohort study, we identified pregnant patients with hepatitis C with linked data to their infants who have had HCV RNA or HCV antibody testing. Demographic data, including age and race/ethnicity, as well as clinical and laboratory data, including tobacco/alcohol use, infections, liver function tests, the HCV RNA titer, HCV antibody, HCV genotype, absolute lymphocyte count, and platelet count, were collected. Data were analyzed using logistic regression and receiver operating characteristics (ROCs) and internally validated using the forward selection bootstrap method. Results: We identified 157 pregnant patients and 163 corresponding infants. The median maternal delivery age was 29 (IQR: 25–33) years, and the majority (141, or 89.8%) were White. A high HCV RNA titer, high absolute lymphocyte count, and high platelet count were associated with vertical transmission. A high HCV RNA titer had an AUROC of 0.815 with sensitivity, specificity, a positive predictive value, and a negative predictive value of 100.0%, 59.1%, 17.6%, and 100.0%, respectively. A composite score combining the three risk factors had an AUROC of 0.902 (95% CI = 0.840–0.964) but with a risk of overfitting. Conclusions: An HCV RNA titer alone or a composite score combining the risk factors for HCV vertical transmission can potentially identify a population of pregnant patients where the rate of vertical transmission is high, allowing for potential interventions during antepartum care.

## 1. Introduction

Globally, there are approximately 71 million persons with chronic hepatitis C, of whom 3.5 million are in the United States [1,2]. Hepatitis C in the United States has been rising over the past two decades and is attributed to the increasing numbers of injection drug users [3]. Similarly, hepatitis C has also been increasing among pregnant women in recent years [4]. A nationwide study from 2009 to 2017 found that hepatitis C virus (HCV) infections occurred in 4.7 cases per 1000 live births in 2017, a significant increase from 1.8 cases per 1000 live births in 2009 [5].

The vertical transmission of HCV from mother to infant is a mode of HCV transmission, leading to significant morbidity in these children. It is estimated that 5–12% of these children develop significant fibrosis, and 5% develop cirrhosis 10–20 years after the initial perinatal infection [6,7].

Overall, the vertical transmission rate of HCV is approximately 5.8% (95% confidence interval (CI) = 4.2–7.8%) [8]. Previous studies identified risk factors increasing the chance of transmission, including coinfection with the human immunodeficiency virus (HIV), which increases the rate of vertical transmission by 2 to 4 fold [9,10,11]. Additionally, the finding of a high HCV RNA titer in previous studies was associated with an increased risk of HCV vertical transmission [10,11,12]. Cesarean section was previously thought to be protective of HCV vertical transmission; however, recent studies were either unable to find significant differences in transmission rates between vaginal and Cesarean delivery or were unclear as to the role of operative delivery regarding a means of protection [10,13,14].

At least one third of the vertical transmission of HCV is thought to occur in utero between 24.9 and 36.1 weeks of gestation [15,16]. As such, any antepartum intervention/treatment may not be effective beyond 36.1 weeks’ gestation and would ideally be initiated before 24.9 weeks’ gestation. Ribavirin was previously used as a common component of HCV treatment regimens, but it is contraindicated in pregnancy (Category X) due to teratogenicity and has now been replaced by modern direct-acting antiviral agents, including ledipasvir and sofosbuvir. To date, these direct-acting antivirals have demonstrated no teratogenic effects. Small Phase 1 clinical trials and case reports using direct-acting antiviral agents to prevent the vertical transmission of HCV, thus far, have found no adverse effects for pregnant patients and fetuses and were effective at preventing the vertical transmission of HCV [17,18]. Understandably, there remains strong hesitancy in the universal treatment of HCV-infected pregnant patients with direct-acting antiviral agents, in large part due to the relatively low transmission rate of 5.8% in comparison to HIV-infected pregnant patients where the vertical transmission rate is 15–45% without treatment, and in hepatitis B virus-infected pregnant patients without treatment, the vertical transmission rate is up to 90% [19,20]. The universal treatment of pregnant patients with hepatitis C would expose approximately 94.2% of them to direct-acting antiviral agents, including any potential adverse effects, without a significant benefit to their fetus/infant.

Our aim is to create a composite score using the risk factors for HCV vertical transmission to identify a population of pregnant patients in which the frequency of vertical transmission is high enough to potentially mitigate the costs and risks of antepartum HCV direct-acting antiviral therapy. To do this, we aim to analyze a cohort of infants exposed to HCV during pregnancy and compare the corresponding maternal characteristics/risk factors between infants that developed HCV infection and infants that did not develop HCV infection.

## 2. Materials and Methods

### 2.1. Study Population

This was a retrospective, multicenter cohort study of infants born from mothers with confirmed hepatitis C during pregnancy. After approval from the Institutional Review Board (IRB) of the University of Florida (IRB# IRB202100598), we screened all patients within the University of Florida Health in Gainesville, FL, and University of Florida Health in Jacksonville, FL, using the following criteria: we included pregnant patients with a diagnosis of hepatitis C who delivered their live-born infants between 1 January 2000 and 24 September 2021. The pregnant patients must have been between 13 and 60 years of age, and they must have had data linking them to their corresponding infants. Infants must have had HCV RNA and/or HCV antibody testing completed by 24 September 2021. Pregnant patients with a diagnosis of hepatitis C after the delivery date were excluded. Similarly, pregnant patients who were diagnosed with hepatitis C before the delivery date and were successfully treated with curative antiviral therapy were excluded. Infants without HCV RNA testing completed at or after two months of age or HCV antibody testing completed at or after 18 months of age were excluded. There were no exclusion criteria for race/ethnicity, socioeconomic status, or number of prior deliveries.

HCV infection in infants was defined based on guidelines from the Centers for Disease Control and Prevention (CDC) and the American Academy of Pediatrics [21,22]. Infants with a positive HCV RNA test at or after two months of age and/or a positive HCV antibody test at or after 18 months of age were considered to be infected with HCV from vertical transmission. Similarly, infants with a negative HCV RNA test at or after two months of age and/or a negative HCV antibody test at or after 18 months of age were considered not to be infected with HCV from vertical transmission. Test results from HCV RNA before two months of age or HCV antibody before 18 months of age were considered inconclusive and not used in this study. If an infant had more than one valid HCV test that was contradictory, the most recent test result was used for the analysis.

### 2.2. Data Extraction

We extracted demographic data, including age at delivery, race/ethnicity of the pregnant patients, and sex of the infant. Additionally, we collected clinical data from the pregnant patients, including alcohol use during pregnancy, tobacco use during pregnancy (including packs smoked per day, years smoked, and pack-years smoked), type 1 or type 2 diabetes mellitus, body mass index (BMI), obesity diagnosis, vaginal bleeding during pregnancy, placental previa, abnormal Papanicolaou (pap) test, history of chlamydia or gonorrhea infection, previous Cesarean section, and Apgar scores. We also collected laboratory data from the pregnant patients, including ABO blood type, Rh type, white blood cell count, absolute lymphocyte count, absolute monocyte count, platelet count, albumin, alanine aminotransferase, aspartate aminotransferase, alkaline phosphatase, total bilirubin, direct bilirubin, HCV antibody status, HCV RNA titer, HCV genotype, hepatitis B virus infection, HIV infection, and herpes simplex infection.

Laboratory data and HCV RNA titers were collected at 20 weeks’ gestation or as close as possible to 20 weeks’ gestation. Pregravid BMI data were scarce in our cohort; thus, we collected BMI data taken at the end of pregnancy as close as possible to the date of delivery.

### 2.3. Statistical Analysis

Continuous data were summarized using the median and interquartile range (IQR). As not all observations had complete data on all the risk factors considered, the risk factors for HCV vertical transmission, including binary and continuous variables, were evaluated individually in a single-predictor logistic regression model, with *p* values calculated using the likelihood ratio test. Risk factors found to be statistically significant at or less than the 5% level were retained, and their estimated effects on HCV vertical transmission were reported as odds ratios (ORs) with 95% confidence intervals (CIs). Odds ratios were derived from exponentiated beta regression coefficients from the logistic regression. Odds ratios from continuous data such as age or ALT represent the increase in odds for each unit increase (i.e., 1 year increase in age or 1 U/L increase in ALT). Control for multiple testing was not made at this stage of the analysis.

Risk factors found to be significant in the single-predictor logistic regression model were then analyzed in a multivariable logistic regression model. The composite score was created from a combination of the significant risk factors from multivariable logistic regression model, and we used the beta coefficients from the multivariable logistic regression model as coefficients in the composite score equation. Significant risk factors from the initial logistic regression and the composite score were evaluated for predictive capacity using ROC curve analysis. Results were reported by area under the receiver operating characteristic curve (AUROC) value, with 95% CI, standard error, sensitivity, and specificity. There is no single best way to determine cutoffs regarding sensitivity and specificity; for this study, we determined optimized cutoffs as the point that resulted in the highest summation of sensitivity and specificity.

As multiple testing was not controlled for in the variable selection process, there is a risk of overfitting. To explore this possibility, a bootstrap analysis was subsequently performed. As the HCV RNA titer was highly statistically significant and because its relevance has also been established in previous studies, the HCV RNA titer was preserved in the bootstrap replications, while the values of each of the remaining risk factors were randomly permuted before applying the model-fitting procedure described above to the resulting bootstrap dataset. This procedure was repeated 10,000 times, and for each of these replications, the risk factors selected were recorded along with the observed AUROC, specificity, sensitivity, positive predictive value, and negative predictive value. For the bootstrap datasets, the selection of any risk factor other than the HCV RNA titer constitutes overfitting in the bootstrap world. For analytical purposes, missing data were assumed to be randomly distributed without significant contributions to the variables evaluated.

Statistical calculations were performed using R program (R Core Team (2020). R: A language and environment for statistical computing. R Foundation for Statistical Computing, Vienna, Austria. URL https://www.R-project.org/, accessed on 5 August 2023).

## 3. Results

Out of 742 pregnant patients with hepatitis C with matched infant data, 193 pregnant patients, with 210 corresponding infants, met the selection criteria. Out of those 193 pregnant patients, 36 (18.7%) had positive HCV antibodies but negative HCV RNA and were excluded from the main analysis. The final cohort included 157 pregnant patients and their corresponding 163 infants, including 6 pairs of twins. The median age at delivery of the pregnant patients was 29 (IQR: 25–33) years of age. The race distribution was 141 (89.8%) White, 6 (3.8%) Black, and 10 (6.4%) Asian/unknown. Of the 163 infants, 90 (55.2%) were female, and 73 (44.8%) were male. Among the 163 infants, the vertical transmission of HCV occurred in 12 (7.4%). The median gestation age that laboratory data was collected from was 28 (IQR: 19–35) weeks (Table 1).

Using logistic regression, we evaluated variables that influence the odds of HCV vertical transmission. A high HCV RNA titer (continuous) was found to be associated with increased odds of HCV vertical transmission (OR = 5.18; 95 CI = 1.95–17.00; *p* < 0.001). Additionally, high absolute lymphocyte counts (continuous) were found to be associated with increased odds of HCV vertical transmission (OR = 3.74; 95% CI = 1.24–12.70; *p* = 0.020). A high platelet count (continuous) was also found to be associated with increased odds of HCV vertical transmission (OR = 2.42; 95% CI = 1.07–5.70; *p* = 0.034). Demographic variables, including age at delivery, race of the mother, and sex of the infant, were not associated with HCV vertical transmission. Additionally, clinical and laboratory variables, including the use of alcohol or tobacco during pregnancy, diabetes, high BMI, vaginal bleeding, placental previa, a history of abnormal pap smear, a history of chlamydia/gonorrhea infection, previous cesarean section, ABO blood type, Rh type, white blood cell count, absolute monocyte count, albumin, alanine aminotransferase, aspartate aminotransferase, alkaline phosphatase, total bilirubin, direct bilirubin, HCV genotype, hepatitis B virus infection, HIV infection, and herpes simplex infection, were not associated with HCV vertical transmission (Table 2 and Table 3).

ROC curves were constructed for each significant continuous risk factor, and the composite score was created from those risk factors to quantify the scores’ effectiveness. Among the risk factors, the HCV RNA titer had the highest AUROC at 0.815 (95% CI = 0.730–0.899), followed by the platelet count at 0.709 (95% CI = 0.570–0.849), and then the absolute lymphocyte count at 0.688 (95% CI = 0.530–0.845). The composite score was found to be superior, with an AUROC of 0.902 (95% CI = 0.840–0.964). The sensitivity and specificity of the composite score were 100.0% and 85.2%, respectively (Table 4). A visual representation of the ROC curves is shown in Supplementary Figure S1. The HCV RNA titer alone, using the cutoff of ≥6.05 logIU/mL, identified 68 pregnant patients who were above the cutoff, of whom 12 (17.6% (positive predictive value)) transmitted HCV to their respective infants; the negative predictive value was 100.0%. Applying the composite score to our cohort and using the cutoff of ≥12.91, we identified 22 pregnant patients who were above the cutoff, of whom 9 (40.9% (positive predictive value)) transmitted HCV to their respective infants. None of the pregnant patients with scores below our cutoff transmitted HCV; the negative predictive value was 100.0%. The composite score was calculated using the formula below:

Composite Score = [HCV RNA Titer Log IU/mL] × 1.273238 + [Lymphocyte Absolute Thou/mL] × 1.236126 + [Platelet Count Thou/mL] × 0.008201

**Figure 1:**
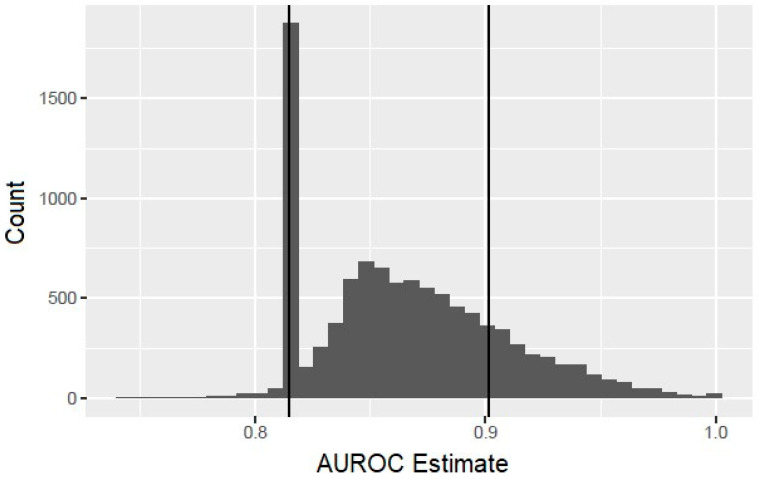
Histogram of bootstrapped area under the receiver operating characteristic curve (AUROC) estimates.

The bootstrap analysis offered some caution with the interpretation of the results. Although in the context of the bootstrap samples, only the HCV RNA titer is predictive of infant HCV, in 82% of the bootstrap replications, the fitting procedure, nevertheless, selected at least one risk factor in addition to the HCV titer, indicating a high probability of overfitting. In addition, the observed performance characteristics of these overfitted models tended to suggest a substantial improvement over the simpler model with the HCV RNA titer as the only predictor. Figure 1 displays a histogram of the bootstrapped AUROC estimates. The vertical line at 0.815 corresponds to the AUROC for the model using only the HCV titer, and the bar centered at that value corresponds to those bootstrap replications for which the model-fitting procedure correctly selected this model. The bars to the right of this correspond to bootstrap replications in which the procedure overfits the data and hence yielded an exaggerated estimate of the AUROC. The additional vertical line at 0.902 corresponds to the AUROC value obtained with the composite score. This value was exceeded in 19.4% of the bootstrap replications, showing that it would not be unusual to achieve such a large value by overfitting the data, even when the additional risk factors added to the model are potentially not associated with the vertical transmission of HCV.

Of note, none of the 47 infants delivered from the 36 pregnant patients with a positive HCV antibody test result but negative HCV RNA subsequently became infected with HCV. Additionally, 4 (2.5%) of the 157 pregnant patients in the final cohort had cirrhosis, none of whom transmitted HCV to their infants.

## 4. Discussion

### 4.1. Principal Findings

We propose a system to stratify HCV vertical transmission risk. We identified three risk factors that significantly increased the odds of HCV vertical transmission: the HCV RNA titer, absolute lymphocyte count, and platelet count. And by integrating all three risk factors, we have created a composite score that is highly sensitive and specific for predicting HCV vertical transmission, with an AUROC of 0.902, a sensitivity of 100.0%, and a specificity of 85.2%, superior to any of the three risk factors alone.

### 4.2. Results in the Context of What Is Known

The HCV RNA titer has been shown to be strongly associated with HCV vertical transmission in several studies, and this is consistent with the results of this study [10,11,12,23,24]. The absolute lymphocyte count and platelet count have not previously been reported to be associated with HCV vertical transmission, but those risk factors were not commonly studied [23]. A high absolute lymphocyte count may be a signal of more active disease regardless of the HCV RNA titer. A study by Nelson et al. reported that patients with HCV-specific cytotoxic T lymphocyte activity had lower levels of viremia but evidence of more active disease [25]. Additionally, the platelet count is known to be an acute phase reactant and may represent increased inflammation in those pregnant patients, leading to increased vascular permeability and a potentially increased risk of mother–fetal transmission. In our study, HIV was not found to be associated with increased odds of HCV vertical transmission, although our patients were typically treated. Previous studies have found HIV to be associated with an increased risk of HCV vertical transmission but inconsistently [9,10,11,23]. A recent study by Garcia-Tejedor et al. investigated risk factors for HCV vertical transmission, including HIV, in a cohort of 710 pregnant patients with hepatitis C and found no association between HIV and an increased risk of HCV vertical transmission [23]. This may be attributed to antiviral HIV suppression.

### 4.3. Clinical and Research Implications

Studies on the antepartum antiviral treatment of pregnant patients with hepatitis C have been limited to small clinic trials, case reports, and case series. A case report by Mandimika and Ogbuagu found no adverse events in sofosbuvir monotherapy for 6 weeks during pregnancy starting at 34 weeks’ gestation, and the infant was confirmed to not be infected with HCV [18]. A Phase 1 clinical trial by Chappell et al. of a 12-week sofosbuvir plus ledipasvir regimen in eight pregnant patients with hepatitis C at 23–24 weeks of gestation found adverse events to be minor, consisting of nausea/vomiting, diarrhea, and fatigue in 56% of patients [17]. Notably, all eight infants in that series were found to be HCV RNA negative at the 12-month follow-up [17]. As at least one third of the vertical transmission of HCV is thought to occur in utero and with the in utero transmission predicted to occur between 24.9 and 36.1 weeks of gestation, antiviral treatment would ideally be initiated before 24.9 weeks’ gestation [15,16]. As such, we chose to collect laboratory data, as well as HCV RNA titers, at 20 weeks of gestation in order to validate our predictive composite score for use at 20 weeks of gestation, giving clinicians time to formulate a treatment plan. Correspondingly, we chose not to include the delivery mode in the development of the composite score as this would not be applicable to antepartum intervention.

Infants born to patients with hepatitis C are at an increased risk of preterm birth, low birth weight, and other congenital anomalies [26]. Infection of the fetus/infant with HCV confers significant morbidity, including liver fibrosis, cirrhosis, liver failure, glomerulonephritis, developmental delays, and cognitive deficits [27]. As HCV infection in fetuses/infants may be asymptomatic for years, liver damage may occur surreptitiously, and these individuals may unknowingly transmit HCV in adolescence or adulthood. While treatment of HCV is approved for children as young as 3 years of age, the lack of follow-up compliance is a major barrier to care. In a study of postpartum follow-up care for pregnant patients with hepatitis C, Jarlenski et al. found that only 19.1% of those diagnosed with hepatitis C returned for follow-up care/treatment within 6 months postpartum [28]. Those infected with hepatitis C may have socioeconomic challenges, limiting access to the health care system outside of pregnancy. The treatment of hepatitis C during pregnancy presents an opportunity to avoid the need for follow-up hepatitis C treatment for the infant at 3 years of age, as well as offering the patient with hepatitis C an opportunity to start direct-acting antiviral therapy during pregnancy, potentially shortening the time of any postpartum curative HCV treatment [18].

### 4.4. Strengths and Limitations

Our study offers a means of identifying prenatal patients at risk of the vertical transmission of HCV, allowing a population for further study to be targeted for antenatal treatment. Further, this methodology avoids exposing those unlikely to transmit HCV to anti-viral trials. Cost-effectiveness is an important consideration of any predictive test or scoring system. The composite score created in this study used data from the routine labs expected from a pregnant woman with hepatitis C. In a pregnant woman known to have hepatitis C, an HCV RNA titer test is likely to be ordered during the pregnancy period. The absolute lymphocyte count and platelet count can be derived from a complete blood count using a differential test which is a common prenatal lab.

**Figure 2:**
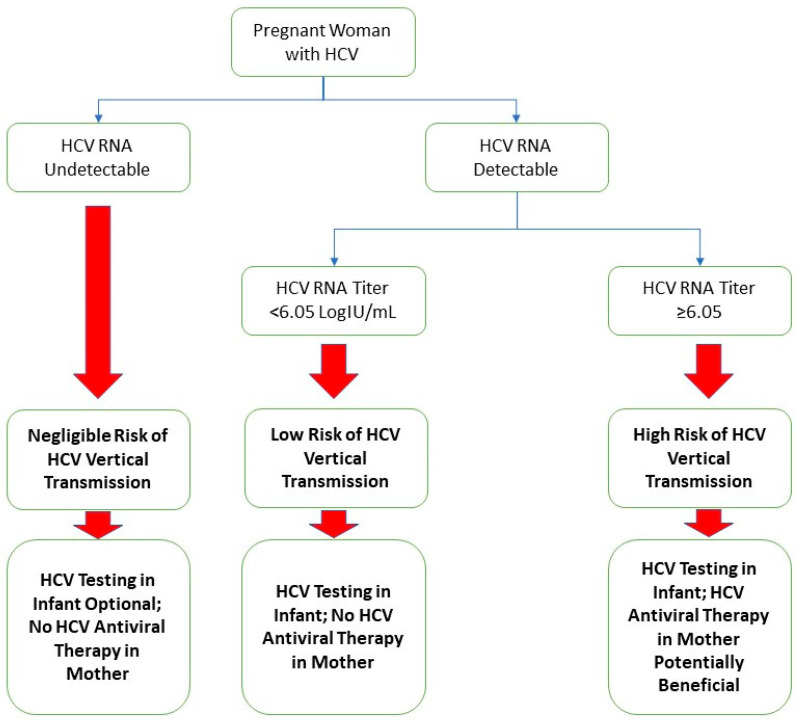
Suggested algorithm for a framework of study regarding pregnant patients with hepatitis C and their infants.

A limitation of this study was the relatively small cohort size. Despite using data from two centers, pregnant patients with hepatitis C were relatively scarce, and infants who received appropriate screening tests for hepatitis C were even less common. The sample size constraints could potentially have led to biases in the development of the composite score, such as incorrect weights for the variables of the composite score, as well as exclusions of truly significant variables that require a larger sample size to appreciate. Another limitation included the retrospective nature of this study and its inherent weaknesses. One of these is the completeness of data; while most patients in our cohort had complete data, there were some patients with missing data in specific variables, reducing the applicability of our analysis. We managed missing data with the assumption that those missing data points were randomly distributed. Another limitation is that when we analyzed the results using the bootstrap method, the results suggested that the apparent improvements in the AUROC and specificity gained by the addition of the absolute lymphocyte count and platelet count are potentially an artifact of the overfitting of the model. Nevertheless, the HCV RNA titer at our optimized cutoff of ≥6.05 logIU/mL maintains a positive predictive value of 17.6%, an improvement of three-fold from the base transmission rate of 5.8% [8]. Additionally, with a negative predictive value of 100.0%, the HCV RNA titer has the potential to be used as a “rule-out” test for HCV vertical transmission. In Figure 2, we provided our proposed algorithm for stratifying the HCV vertical transmission risk and management decisions of pregnant patients with hepatitis C based on the results of this study.

## 5. Conclusions

While the antepartum antiviral treatment of hepatitis C in pregnant patients is reportedly safe in a few published cases, the practice is not widespread considering the relatively low rate of HCV vertical transmission. A high HCV RNA titer is associated with HCV vertical transmission, and an HCV RNA titer < 6.05 logIU/mL has an excellent negative predictive value. Although limited by the sample size, this preliminary study demonstrates the groundwork for a composite score based on risk factors for HCV vertical transmission to identify a population of high-risk pregnant patients as potential candidates for antiviral treatment. A composite score can stratify pregnant women with HCV infection into high- and low-risk groups, helping clinicians determine who may need close monitoring and follow-up for their infants, as well as those who can potentially benefit from antiviral treatment during pregnancy and who can be treated after delivery. A future study with a larger, independent cohort investigating the significant risk factors (i.e., the HCV RNA titer, platelet count, and absolute lymphocyte count) or comparing the performance of our composite score to the HCV RNA titer alone would be beneficial.

## Figures and Tables

**Table 1:** Summary of maternal demographic, clinical, and laboratory characteristics.

Characteristics	Overall	Positive HCV Transmission	Negative HCV Transmission
Total Cases	163	12	151
*Demographics*			
Age, Year, Median (IQR)	29 (25–33)	30 (26–32)	28 (25–33)
Race			
White, N (%)	146 (89.6%)	11 (91.7%)	135 (89.4%)
Black, N (%)	6 (3.7%)	1 (8.3%)	5 (3.3%)
Other, N (%)	11 (6.7%)	0 (0.0%)	11 (7.3%)
Sex of Infant			
Female, N (%)	90 (55.2%)	6 (50.0%)	84 (55.6%)
Male, N (%)	73 (44.8%)	6 (50.0%)	67 (44.4%)
*Clinical*			
Alcohol Use, N (%)	27 (16.6%)	1 (8.3%)	26 (17.2%)
Tobacco Use, N (%)	128 (78.5%)	10 (83.3%)	118 (78.1%)
Tobacco Packs Per Day, Median (%)	0.5 (0.5–1.0)	1.0 (0.4–1.5)	0.5 (0.5–1.0)
Diabetes, N (%)	31 (19.0%)	2 (16.7%)	29 (19.2%)
BMI, kg/m^2^, Median (IQR)	27.6 (25.9–31.4)	28.8 (26.1–34.3)	27.6 (25.9–31.2)
Vaginal Bleeding, N (%)	27 (16.7%)	1 (8.3%)	26 (17.2%)
Placental Previa, N (%)	9 (5.5%)	1 (8.3%)	8 (5.3%)
Abnormal Pap Smear, N (%)	28 (17.2%)	3 (25.0%)	25 (16.6%)
History of Chlamydia/Gonorrhea, N (%)	19 (11.7%)	1 (8.3%)	18 (11.9%)
Previous Cesarean Section, N (%)	42 (25.8%)	2 (16.7%)	40 (26.5%)
*Laboratory*			
ABO Blood Type			
A Type, N (%)	69 (42.3%)	5 (41.7%)	64 (42.4%)
B Type, N (%)	19 (11.7%)	0 (0.0%)	19 (12.6%)
O Type, N (%)	71 (43.6%)	7 (58.3%)	64 (42.4%)
Rh Type Positive, N (%)	137 (84.0%)	10 (83.3%)	127 (84.1%)
White Blood Cell, 10^3^/mL, Median (IQR)	9.7 (7.7–12.1)	10.0 (9.5–11.8)	9.6 (7.6–12.1)
Lymphocyte Absolute, 10^3^/mL, Median (IQR)	1.9 (1.5–2.3)	2.2 (2.0–2.2)	1.9 (1.5–2.3)
Monocyte Absolute, 10^3^/mL, Median (IQR)	0.5 (0.4–0.7)	0.6 (0.6–0.9)	0.5 (0.4–0.7)
Platelet, 10^3^/mL, Median (IQR)	240 (194–289)	290 (240–329)	237 (191–276)
Albumin, g/dL, Median (IQR)	3.5 (3.2–3.8)	3.6 (3.1–3.8)	3.5 (3.2–3.8)
ALT, U/L, Median (IQR)	23 (16–38)	18 (17–60)	23 (16–38)
AST, U/L, Median (IQR)	24 (18–37)	27 (23–48)	24 (18–33)
ALP, U/L, Median (IQR)	84 (61–140)	70 (64–109)	84 (61–156)
Bilirubin Total (mg/dL), Median (IQR)	0.3 (0.2–0.4)	0.3 (0.2–0.4)	0.3 (0.2–0.4)
Bilirubin Direct (mg/dL), Median (IQR)	0.1 (0.0–0.1)	0.1 (0.0–0.1)	0.1 (0.0–0.1)
HCV Titer, Log IU/mL, Median (IQR)	5.92 (5.32–6.43)	6.66 (6.28–6.90)	5.85 (5.29–6.37)
HCV Genotype			
Genotype 1, N (%)	58 (35.6%)	6 (50.0%)	52 (34.4%)
Genotype 2, N (%)	18 (11.0%)	2 (16.7%)	16 (10.6%)
Genotype 3, N (%)	24 (14.7%)	1 (8.3%)	23 (15.2%)
HBV Infection, N (%)	10 (6.1%)	0 (0.0%)	10 (6.6%)
HIV Infection, N (%)	7 (4.3%)	0 (0.0%)	7 (4.6%)
Herpes Simplex Infection, N (%)	21 (12.9%)	0 (0.0%)	21 (13.9%)

ALP = alkaline phosphatase; ALT = alanine aminotransferase; AST = aspartate aminotransferase; BMI = body mass index; HBV = hepatitis B virus; HCV = hepatitis C virus; HIV = human immunodeficiency virus; IQR = interquartile range; Rh = rhesus.

**Table 2:** Likelihood ratio test of risk factors for association with hepatitis C vertical transmission.

	Risk Factor		*p* Value
*Demographics*			
	Age (Year)	Quantitative	0.648
	Race	White/Black/Other	0.799
	Sex of Infant	Female/Male	0.707
*Clinical*			
	Alcohol Use	Yes/No	0.646
	Tobacco Use	Yes/No	0.602
	Tobacco Packs Per Day	Quantitative	0.328
	Diabetes	Yes/No	0.697
	BMI (kg/m^2^)	Quantitative	0.643
	Vaginal Bleeding	Yes/No	0.389
	Placental Previa	Yes/No	0.677
	Abnormal Pap Smear	Yes/No	0.476
	History of Chlamydia/Gonorrhea	Yes/No	0.697
	Previous Cesarean Section	Yes/No	0.434
*Laboratory*			
	ABO Blood Type	A/B/O	0.250
	Rh Type	Yes/No	0.944
	White Blood Cell (10^3^/mL)	Quantitative	0.986
	Lymphocyte Absolute (10^3^/mL)	Quantitative	**0.019**
	Monocyte Absolute (10^3^/mL)	Quantitative	0.247
	Platelet (10^3^/mL)	Quantitative	**0.034**
	Albumin (g/dL)	Quantitative	0.888
	ALT (U/L)	Quantitative	0.810
	AST (U/L)	Quantitative	0.963
	ALP (U/L)	Quantitative	0.170
	Bilirubin Total (mg/dL)	Quantitative	0.689
	Bilirubin Direct (mg/dL)	Quantitative	0.375
	HCV Titer (Log IU/mL)	Quantitative	**<0.001**
	HCV Genotype	1/2/3	0.699
	HBV	Yes/No	0.209
	HIV	Yes/No	0.295
	Herpes Simplex	Yes/No	0.063

ALP = alkaline phosphatase; ALT = alanine aminotransferase; AST = aspartate aminotransferase; BMI = body mass index; HBV = hepatitis B virus; HCV = hepatitis C virus; HIV = human immunodeficiency virus; Rh = rhesus. Bolded *p*-values are statistically significant.

**Table 3:** Multivariable logistic regression with odds ratio of significant risk factors from likelihood ratio test.

Risk Factor		Odds Ratio (95% CI)	*p* Value
Lymphocyte Absolute (10^3^/mL)	High (vs. Low)—Quantitative	3.74 (1.24–12.70)	**0.020**
Platelet (10^5^/mL)	High (vs. Low)—Quantitative	2.42 (1.07–5.70)	**0.034**
HCV Titer (Log IU/mL)	High (vs. Low)—Quantitative	5.18 (1.95–17.00)	**<0.001**

HCV = hepatitis C virus. Bolded *p*-values are statistically significant.

**Table 4:** AUROC with sensitivity and specificity of significant risk factors for hepatitis C vertical transmission and the resulting composite score.

Scoring System	AUROC Curve (95% CI)	Standard Error	Optimized Cutoff	Sensitivity	Specificity	PPV	NPV
HCV Titer (Log IU/mL)	0.815 (0.730–0.899)	0.043	≥6.05	100.0%	59.1%	17.6%	100.0%
Lymphocyte Absolute (10^3^/mL)	0.688 (0.530–0.845)	0.080	≥1.86	88.9%	46.6%	14.3%	97.6%
Platelet (10^3^/mL)	0.709 (0.570–0.849)	0.071	≥235	91.7%	49.0%	12.5%	98.7%
Composite Score	0.902 (0.840–0.964)	0.032	≥12.91	100.0%	85.2%	40.9%	100.0%

AUROC = area under receiver operating characteristic curve; CI = confidence interval; HCV = hepatitis C virus; PPV = positive predictive value; NPV = negative predictive value.

## Data Availability

The data presented in this study are available on request from the corresponding author. The data are not publicly available due to privacy or ethical restrictions.

## Supplementary Materials

The following supporting information can be downloaded at: https://www.mdpi.com/article/10.3390/pathogens13010045/s1, Figure S1: Receiver operating characteristic curves of significant risk factors for hepatitis C vertical transmission and the resulting composite score.

## References

[1] World Health Organization (2017). Global Hepatitis Report 2017.

[2] Hofmeister M.G., Rosenthal E.M., Barker L.K., Rosenberg E.S., Barranco M.A., Hall E.W., Edlin B.R., Mermin J., Ward J.W., Ryerson A.B. (2019). Estimating Prevalence of Hepatitis C Virus Infection in the United States, 2013–2016. Hepatology.

[3] Zibbell J.E., Asher A.K., Patel R.C., Kupronis B., Iqbal K., Ward J.W., Holtzman D. (2018). Increases in Acute Hepatitis C Virus Infection Related to a Growing Opioid Epidemic and Associated Injection Drug Use, United States, 2004 to 2014. Am. J. Public Health.

[4] Schillie S.F., Canary L., Koneru A., Nelson N.P., Tanico W., Kaufman H.W., Hariri S., Vellozzi C.J. (2018). Hepatitis C Virus in Women of Childbearing Age, Pregnant Women, and Children. Am. J. Prev. Med..

[5] Rossi R.M., Wolfe C., Brokamp R., McAllister J.M., Wexelblatt S., Warshak C.R., Hall E.S. (2020). Reported Prevalence of Maternal Hepatitis C Virus Infection in the United States. Obstet. Gynecol..

[6] Mohan P., Colvin C., Glymph C., Chandra R.R., Kleiner D.E., Patel K.M., Luban N.L.C., Alter H.J. (2007). Clinical Spectrum and Histopathologic Features of Chronic Hepatitis C Infection in Children. J. Pediatr..

[7] Guido M., Bortolotti F., Leandro G., Jara P., Hierro L., Larrauri J., Barbera C., Giacchino R., Zancan L., Balli F. (2003). Fibrosis in chronic hepatitis C acquired in infancy: Is it only a matter of time?. Am. J. Gastroenterol..

[8] Benova L., Mohamoud Y.A., Calvert C., Abu-Raddad L.J. (2014). Vertical transmission of hepatitis C virus: Systematic review and meta-analysis. Clin. Infect. Dis..

[9] Polis C.B., Shah S.N., Johnson K.E., Gupta A. (2007). Impact of maternal HIV coinfection on the vertical transmission of hepatitis C virus: A meta-analysis. Clin. Infect. Dis..

[10] Mariné-Barjoan E., Berrébi A., Giordanengo V., Favre S.F., Haas H., Moreigne M., Izopet J., Tricoire J., Tran A., Pradier C. (2007). HCV/HIV co-infection, HCV viral load and mode of delivery: Risk factors for mother-to-child transmission of hepatitis C virus?. AIDS.

[11] Cottrell E.B., Chou R., Wasson N., Rahman B., Guise J.M. (2013). Reducing risk for mother-to-infant transmission of hepatitis C virus: A systematic review for the U.S. preventive services task force. Ann. Intern. Med..

[12] Mast E.E., Hwang L.Y., Seto D.S.Y., Nolte F.S., Nainan O.V., Wurtzel H., Alter M.J. (2005). Risk factors for perinatal transmission of hepatitis C virus (HCV) and the natural history of HCV infection acquired in infancy. J. Infect. Dis..

[13] Gibb D.M., Goodall R.L., Dunn D.T., Healy M., Neave P., Cafferkey M., Butler K. (2000). Mother-to-child transmission of hepatitis C virus: Evidence for preventable peripartum transmission. Lancet.

[14] Ghamar Chehreh M.E., Tabatabaei S.V., Khazanehdari S., Alavian S.M. (2011). Effect of cesarean section on the risk of perinatal transmission of hepatitis C virus from HCV-RNA+/HIV-mothers: A meta-analysis. Arch. Gynecol. Obstet..

[15] Fauteux-Daniel S., Larouche A., Calderon V., Boulais J., Béland C., Ransy D.G., Boucher M., Lamarre V., Lapointe N., Boucoiran I. (2017). Vertical Transmission of Hepatitis C Virus: Variable Transmission Bottleneck and Evidence of Midgestation In Utero Infection. J. Virol..

[16] Mok J., Pembrey L., Tovo P.A., Newell M.L. (2005). When does mother to child transmission of hepatitis C virus occur?. Arch. Dis. Child. Fetal Neonatal Ed..

[17] Chappell C.A., Scarsi K.K., Kirby B.J., Suri V., Gaggar A., Bogen D.L., Macio I.S., Meyn L.A., Bunge K.E., Krans E.E. (2020). Ledipasvir plus sofosbuvir in pregnant women with hepatitis C virus infection: A phase 1 pharmacokinetic study. Lancet Microbe.

[18] Mandimika C., Ogbuagu O. (2019). Successful sofosbuvir lead-in monotherapy for the treatment of hepatitis C virus (HCV) infection in a pregnant woman living with HIV. BMJ Case Rep..

[19] Barral M.F.M., de Oliveira G.R., Lobato R.C., Mendoza-Sassi R.A., Martínez A.M.B., Gonçalves C.V. (2014). Risk factors of HIV-1 vertical transmission (VT) and the influence ofantiretroviral therapy (ART) in pregnancy outcome. Rev. Inst. Med. Trop. Sao Paulo.

[20] Gentile I., Borgia G. (2014). Vertical transmission of hepatitis B virus: Challenges and solutions. Int. J. Women’s Health.

[21] Schillie S., Wester C., Osborne M., Wesolowski L., Ryerson A.B. (2020). CDC recommendations for hepatitis C screening among adults-United States, 2020. MMWR Recomm. Rep..

[22] Kimberlin D., Barnett E., Lynfield R., Sawyer M., American Academy of Pediatrics (2021). Hepatitis C. Red Book: 2021 Report of the Committee on Infectious Diseases.

[23] Garcia-Tejedor A., Maiques-Montesinos V., Diago-Almela V.J., Pereda-Perez A., Alberola-Cuñat V., López-Hontangas J.L., Perales-Puchalt A., Perales A. (2015). Risk factors for vertical transmission of hepatitis C virus: A single center experience with 710 HCV-infected mothers. Eur. J. Obstet. Gynecol. Reprod. Biol..

[24] Oliver E.A., Waterman E., Kuncio D., Addish E., Fenkel J., Tholey D., Schuster M. (2022). Maternal viral load and risk of vertical transmission in patients with Hepatitis C. Am. J. Obstet. Gynecol..

[25] Nelson D.R., Marousis C.G., Davis G.L., Rice C.M., Wong J., Houghton M., Lau J.Y. (1997). The role of hepatitis C virus-specific cytotoxic T lymphocytes in chronic hepatitis C. J. Immunol..

[26] Connell L.E., Salihu H.M., Salemi J.L., August E.M., Weldeselasse H., Mbah A.K. (2011). Maternal hepatitis B and hepatitis C carrier status and perinatal outcomes. Liver Int..

[27] Mack C.L., Gonzalez-Peralta R.P., Gupta N., Leung D., Narkewicz M.R., Roberts E.A., Rosenthal P., Schwarz K.B. (2012). NASPGHAN Practice guidelines: Diagnosis and management of hepatitis c infection in infants, children, and adolescents. J. Pediatr. Gastroenterol. Nutr..

[28] Jarlenski M., Chen Q., Ahrens K.A., Allen L., Austin A.E., Chappell C., Donohue J.M., Hammerslag L., Lanier P., McDuffie M.J. (2022). Postpartum Follow-up Care for Pregnant Persons with Opioid Use Disorder and Hepatitis C Virus Infection. Obstet. Gynecol..

