# Supplementary material for: A Composite Score for Predicting Vertical Transmission of Hepatitis C: A Multicenter Study
Source: Pathogens. 2024 Jan 3;13(1):45. doi: 10.3390/pathogens13010045 (PMC10821345; doi:10.3390/pathogens13010045)
Supplement: Supplementary file 1 [file pathogens-13-00045-s001.zip › pathogens-2779728-supplementary.pdf]

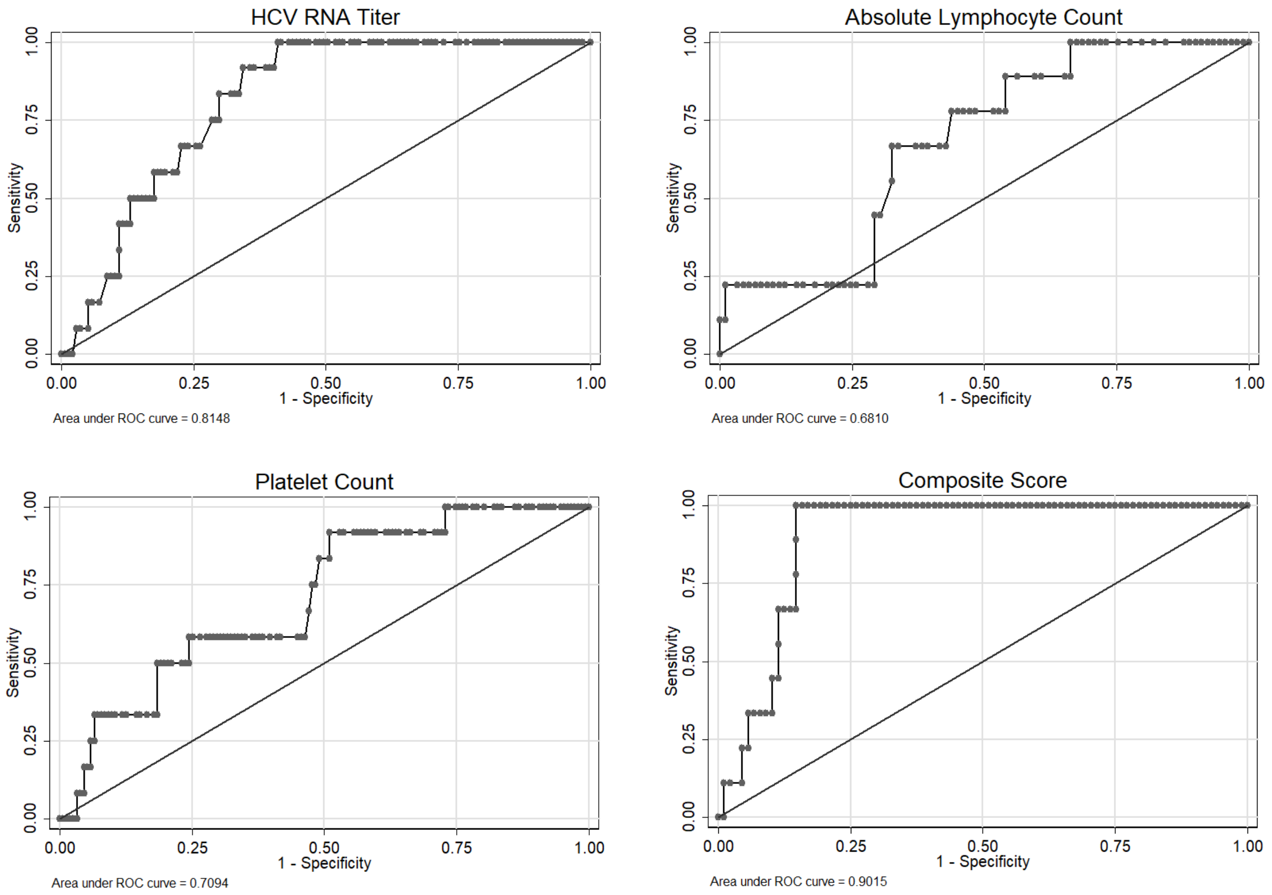

Supplementary Figure S1. Receiver operating characteristic curves of significant risk factors for hepatitis C vertical transmission and the resulting composite score.
